# Protein quality control in the endoplasmic reticulum

**DOI:** 10.1016/j.ceb.2020.04.002

**Published:** 2020-08

**Authors:** Ben P. Phillips, Natalia Gomez-Navarro, Elizabeth A. Miller

**Affiliations:** MRC Laboratory of Molecular Biology, Cambridge, UK

**Keywords:** Protein quality control, Endoplasmic reticulum

## Abstract

Misfolded and mistargeted proteins in the early secretory pathway present significant risks to the cell. A diverse and integrated network of quality control pathways protects the cell from these threats. We focus on the discovery of new mechanisms that contribute to this protective network. Biochemical and structural advances in endoplasmic reticulum targeting fidelity, and in the redistribution of mistargeted substrates are discussed. We further review new discoveries in quality control at the inner nuclear membrane in the context of orphaned subunits. We consider developments in our understanding of cargo selection for endoplasmic reticulum export. Conflicting data on quality control by cargo receptor proteins are discussed and we look to important future questions for the field.

## Introduction

**Figure 1:**
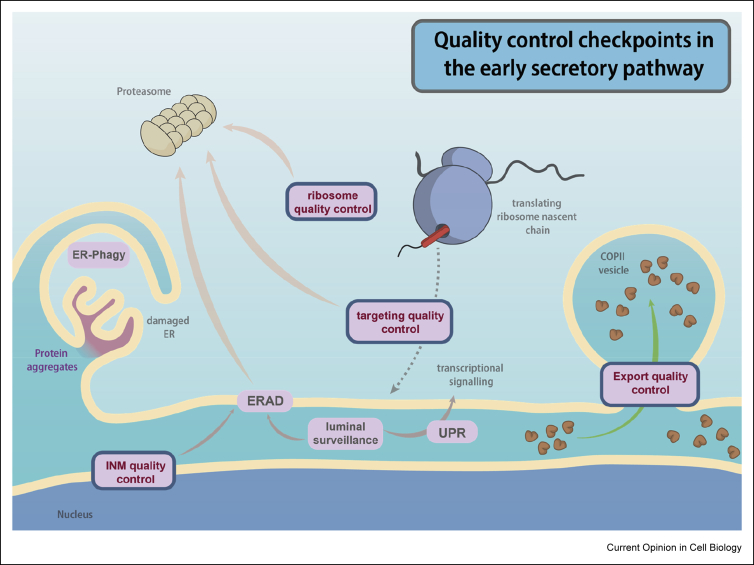
**Quality control checkpoints in the early secretory pathway.** Nascent proteins in the secretory pathway are subject to a multiple quality control checkpoints. These pathways include: degradation of large regions of the ER (ER-phagy); lumenal surveillance of the folding and glycosylation state of nascent proteins; induction of the transcriptional UPR in response to protein folding stress; and ERAD of misfolded and aberrant substrates. In this review, we focus on ribosome-associated quality control of potentially damaging substrates, quality control in the targeting of such substrates and quality control of the export of cargo from the ER. INM, inner nuclear membrane; UPR, unfolded protein response.

Eukaryotic cells have evolved extensive quality control processes that centre on the endoplasmic reticulum (ER) to protect against the accumulation of aberrant secretory and membrane proteins. Collectively, these pathways form a partially redundant network that ensures efficient degradation of substrates with diverse features and localizations. Canonical pathways include ER-associated degradation (ERAD), which extracts proteins from the ER lumen or membrane for proteasomal degradation, and a specialized form of autophagy, ER-phagy, that engulfs parts of the ER that contain aggregated proteins for lysosomal destruction [1,2]. Additional specialized quality control pathways monitor the fidelity of post-translational targeting to organelles, survey the folding status of proteins at early stages during their synthesis, and degrade orphan subunits of multicomponent complexes. Excellent recent reviews have covered some of these diverse quality control pathways [1,3, 4, 5]. Here, we will focus more narrowly on two specific aspects of quality control in the ER: quality control events that monitor protein targeting and integrity early during synthesis, and quality control during ER export via transport vesicles (Figure 1).

## Quality control of organelle targeting: reading the signals right

A long-standing paradox of protein targeting is that proteins that reside in different organelles often possess signal sequences with overlapping physicochemical properties [5]. Indeed, some proteins such as the mitochondrial and peroxisomal fission factor, Mff, can be targeted to more than one organelle in the same cell [6]. Despite potential overlap in signal properties, the proteome of different organelles is tightly controlled and distinct from its neighbours. Such control is achieved both by ensuring initial targeting fidelity as well as by rectifying targeting failure. However, the relative cost of correcting mistargeting increases as the substrate commits to the incorrect path. Thus, maximising fidelity at the earliest stages of targeting, in particular during initial substrate discrimination, is of critical importance and can occur before the nascent chain emerges from the ribosome.

**Figure 2:**
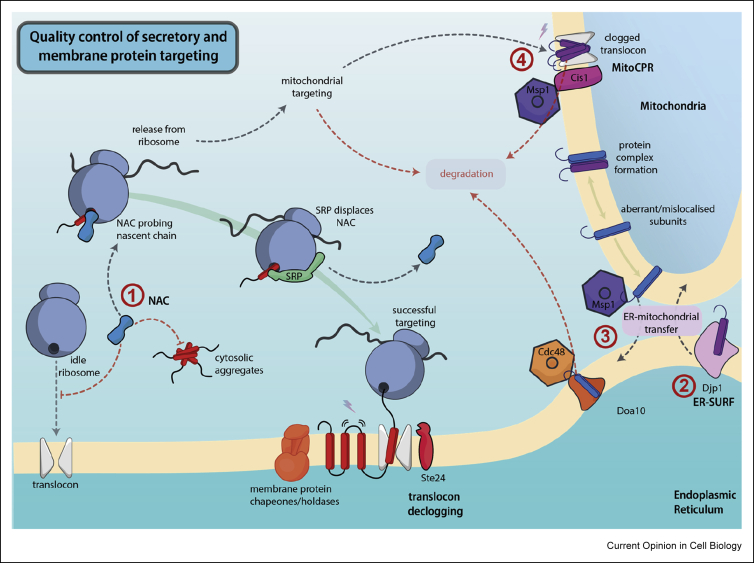
**Quality control of secretory and membrane protein targeting.** (**a**) NAC competes with SRP for binding to the large ribosomal subunit, preventing nonspecific interactions with ER translocons. NAC also acts to prevent cytosolic aggregation independent of its role on the ribosome. (**b**) Mitochondrial membrane proteins that have been incorrectly targeted to the ER are recognised by the ER-SURF pathway and redirected to mitochondria. Failed mitochondrial targeting can also lead directly to degradation as detailed in Ref. [3]. (**c**) Integral membrane proteins subject to quality control in the mitochondria are extracted by Msp1, before being transferred to the ER and being targeted for degradation by Doa10/Cdc48. (**d**) If excess or aberrant substrates reach the translocons of the outer mitochondrial membrane they can cause a clog. This clog is resolved by the actions of Msp1 and the adaptor protein Cis1, as part of the mitochondrial stress response termed ‘MitoCPR’. The declogger Ste24 performs a similar function at the ER.

Since its discovery almost 40 years ago [7], the decision to first target to the ER has been attributed to the signal recognition particle (SRP). However, evidence emerging across the last 10 years builds a more complex model of targeting factors and chaperones that engage with ribosome-nascent chains (RNCs) and serve to sharpen targeting fidelity to both ER and mitochondria [8]. Recent structural and functional work has revealed that the nascent polypeptide-associated complex (NAC) interacts intimately with RNCs and competes with SRP for binding (Figure 2). NAC is thought to act upstream of SRP to prevent improper interactions of RNCs with ER targeting machinery. Specifically, NAC prevents mistargeting at least in part by inhibiting nonspecific interactions with the ER translocon [9]. Disruption of NAC results in mistargeting of mitochondrial proteins to the ER, mirroring the mistargeting of ER substrates to the mitochondria on disruption of SRP [10]. New structures reveal that NAC inserts a ‘probing finger’ into the ribosome exit tunnel to monitor the growing polypeptide chain [11], in contrast to SRP that binds substrates via a domain positioned at the mouth of the exit tunnel, consistent with a later role in targeting [12]. NAC's ‘probing finger’ is essential for function, although the mechanistic details require further clarification, and a potential role for NAC in chaperoning emerging substrates on the ribosome merits additional investigation. A subpopulation of cytosolic NAC has also been shown to protect against cytosolic folding stress, independent of the role for the complex on the ribosome [13], thus NAC likely acts as a multipurpose quality control agent during protein targeting and biogenesis.

Despite the action of SRP, NAC and a variety of kinetic filter steps during membrane protein targeting [5], the shared hydrophobic characteristics of ER and mitochondrial signal sequences can result in targeting errors. These errors must be rectified post-translationally, after organelle targeting. In cases of mitochondrial membrane proteins being mistargeted to the ER, mislocalisation is not necessarily terminal. A novel pathway, termed ‘ER-SURF’ (Figure 2) utilises the chaperone Djp1 to transfer mislocalised mitochondrial proteins away from the ER and facilitate their insertion into the outer mitochondrial membrane [14]. On the other hand, when ER membrane proteins are mislocalised, either to the mitochondria (Figure 2) or the peroxisome, they are extracted and targeted for degradation by the hexametric ATPase Msp1 [15,16]. Recognition of such mislocalised substrates probably involves assessment of oligomeric state [17,18] and detection of characteristic hydrophobic and charged motifs adjacent to the transmembrane domain [19]. Substrates mistargeted to mitochondria are extracted by Msp1 near ER contact sites before ubiquitination and degradation at the ER membrane in a process involving the traditional ERAD components Doa10 and Cdc48 [17,20,21]. These findings emphasise the integrated nature of cellular quality control and exhibit the role of core ERAD machinery in the degradation of aberrant membrane proteins, even when the substrates are found in a separate organelle.

Subsequent to correct targeting, it is now clear that the process of insertion into the bilayer is also subject to quality control. Yeast have evolved systems to resolve translocon clogs (Figure 2), both at the ER via Ste24 [22] and on the mitochondrial outer membrane through the induction of the ‘mitoCPR’ response [23]. Ste24 acts as an intramembrane protease, directly liberating fragments that clog the translocon pore. Conversely, Cis1 recruits the AAA ATPase, Msp1, to clogged translocons, driving extraction and degradation of substrates. Disruption of translocon function in the ER, as well as disruption of the membrane domain insertase EMC can also induce a distinct co-translational quality control response [24] that involves ribosome-associated quality control machinery [4] and translational attenuation. This pre-emptive quality control pathway could restrict the biogenesis of misfolded proteins before they can cause further harm.

Remarkably, even substrates that have been correctly targeted and inserted into the ER membrane can become mislocalised. A complex of Asi1/2/3 in yeast patrols the inner nuclear membrane (INM), which is continuous with, but functionally distinct from, the ER membrane. The Asi complex extracts and degrades mislocalised ER proteins after they diffuse into the INM [25,26]. The Asi complex also serves to remove excess, orphaned subunits that cannot find their cognate partners. Orphaned proteins in the ER are more prone to diffusion into the INM than complete complexes. Here, the orphaned subunits are recognised by Asi2, ubiquitinated and targeted for degradation by Asi1/3 [27]. This spatially restricted degradation provides a pleasing explanation as to how mature orphan subunits are efficiently degraded, whereas newly synthesized subunits in the ER are generally protected from degradation for long enough to find their binding partners. Reminiscent of the Msp1-mediated extraction from the mitochondria for degradation via ERAD, Asi-mediated degradation is also dependent on components shared with conventional ERAD; in this case, Cdc48 and Ubc7/Ubc4 [28]. Figure 2 provides an overview of the discussed mechanisms of quality control in secretory and membrane protein targeting.

Once proteins have been delivered to the ER, chaperones and post-translational modification machineries act to promote folding, which is generally a prerequisite for onward traffic. It is generally thought that this folding stage is where ERAD acts, to detect proteins that fail to fold correctly and shuttle them to the cytoplasmic proteasome system for degradation [1]. The concept that only folded proteins are captured into ER-derived vesicles stems from long-standing empirical observations about the fate of misfolded proteins, but the mechanisms by which such quality control might occur remains poorly understood. Moreover, recent studies suggest that quality control at the level of cargo packaging into vesicles may be more complex.

## Quality control of ER export in the context of protein misfolding

**Figure 3:**
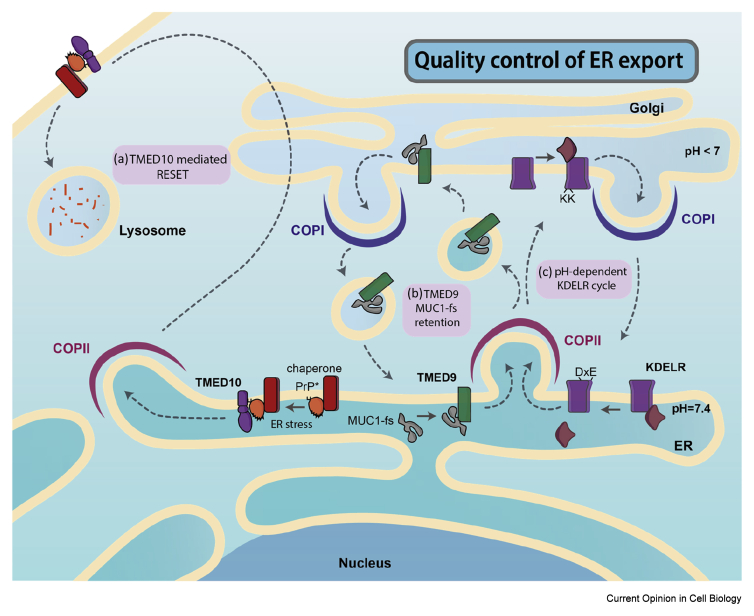
**Recently discovered quality control mechanisms of ER export.** (**a**) RESET of misfolded GPI-APs. ER stress triggers the association of PrP∗ with the cargo receptor TMED10, which enhances export of the misfolded protein in complex with ER chaperones from the ER to the plasma membrane via the Golgi. Once at the plasma membrane, PrP∗ is internalized, and trafficked to the lysosome for degradation. The presence of TMED10 at the plasma membrane is essential for endocytosis and lysosomal degradation of PrP∗. (**b**) Intracellular misfolded mucin, MUC1-fs, accumulation by TMED9. The chimeric toxic Muc1-fs protein is trapped in TMED9-enriched vesicles. Retention of this protein in early secretory compartments prevents trafficking to the lysosome and degradation. (**c**) pH-dependent retrieval of ER proteins. Structural changes of the KDELR triggered by the low pH in the Golgi promote KDEL binding and association with the COPI coat allowing retrograde trafficking of ER residents. The higher pH of the ER triggers cargo protein dissociation and interaction with COPII machinery promoting anterograde trafficking of the receptor.

One obvious mechanism of regulating ER export in the context of protein folding is through cargo receptors. By recruiting cargo for specific capture into COPII vesicles, cargo receptors can monitor the folding status of their clients before capture into vesicles. The full spectrum of cargo receptors that drive ER export remain to be determined, but recent genetic [29] and proteomic [30] approaches are pointing to new candidates that warrant further investigation. Receptor-mediated ER export relies on cargo binding in the donor compartment and release in the acceptor organelle (Figure 3). Although it remains to be fully substantiated, a simple mechanism of ER export receptors only binding folded clients clearly represents a quality control checkpoint that might ensure secretion of folded proteins. Compartment-specific binding and release can be achieved by capitalizing on gradients in lumenal pH and Ca^2+^ concentration between the ER and Golgi. Crystal structures of the KDEL-receptor, which returns escaped ER resident proteins from the *cis*-Golgi back to the ER (Figure 3), revealed exciting molecular details of its pH-dependent binding-release cycle [31]. Changes in pH not only regulate cargo binding but also trigger conformational changes in the cargo receptor so it exposes a COPI binding motif at the Golgi and a putative COPII sorting signal at the ER. Thus, orchestrated conformational changes in the receptor mediated by pH drive the retrieval of escaped ER residents and allow the receptor's shuttling between the Golgi and the ER. Whether similar context-dependent conformational changes occur in productive cargo-driven ER export awaits structural analysis of ER export receptors.

Another emerging layer of quality control conferred by cargo receptors is to prevent oligomerization of secretory proteins that assemble into larger complexes in later compartments. Various dentin-associated proteins oligomerize in the extracellular matrix, triggered by high protein concentration and calcium conditions that mirror that of the ER. During transit through the ER, these proteins are prevented from accumulating by rapidly engaging the ER export receptor, SURF4, which promotes their exit from the ER and maintains them at subaggregation concentrations [32].

A separate example for the broader roles cargo receptors play in quality control is that of the p24 proteins, best characterized in yeast as ER export receptors for glycosylphosphatidylinositol-anchored proteins [33,34]. In yeast and worms, mutation of p24 proteins increases the ER export of misfolded proteins and ER residents [35,36]. Similarly, in animal cells, depletion of p24 proteins from COPII vesicles by abrogating interaction with the COPII machinery results in a five-fold increase in packaging of ER residents [37]. How this family of cargo receptors contributes to the specificity of ER export remains to be fully explained, including whether the cargo-binding activity is required for the sorting stringency phenotype. One model for p24-dependent sorting stringency posits that p24 proteins oligomerise into an array within the nascent vesicle that excludes resident proteins in the lumen of the ER [37].

A broader role for the p24 family of proteins in ER export quality control is emerging from examples where ER sorting stringency lapses. One example is rapid ER stress-induced export, or RESET (Figure 3), which seems to clear misfolded GPI-anchored proteins (such as PrP∗) from the ER under acute stress conditions [38]. On RESET activation, PrP∗, in complex with ER chaperones and the p24 protein TMED10, leaves the ER and transits through the secretory pathway to the plasma membrane, where it is internalised for degradation in the lysosome (Figure 3). The presence of ER chaperones and TMED10 bound to PrP∗ at the plasma membrane is critical for lysosomal targeting, as in their absence PrP∗ is not internalised for degradation [39]. In this case, ER chaperones and cargo receptors play dual roles: preventing the engagement of PrP∗ with ERAD, and driving internalization and lysosomal delivery from the plasma membrane. Further complexity in p24 family function comes from an additional role in quality control of mutant mucin, MUC1-fs. Lysosomal delivery of this misfolded protein is prevented by the p24 protein TMED9, which seems to retain MUC1-fs in early secretory compartments (Figure 3). On TMED9 release, triggered by a small molecule, MUC1-fs travels though the secretory pathway to the lysosome for degradation [40]. Understanding the diverse roles this family of proteins is playing in cargo handling requires deeper insight into the mechanisms of cargo binding, and how such interactions impact engagement with various sorting machineries.

## Conclusions

Protein quality control within the ER operates on many different levels, but universally relies on the molecular recognition of defective proteins. Generally, investigation of these recognition events has focused on the physical state of the protein, including properties like exposure of hydrophobic patches, protein aggregation, and glycan trimming. As we gain more detailed insight into the molecular networks that make quality control decisions, it is apparent that additional parameters are also important. In the past 5 years, the environmental context of potential substrates has emerged as an important fate determinant. Some of the examples highlighted in this review demonstrate how contextual information is integrated to bias substrate fates from probiogenesis to competing retargeting or degradative pathways. Orphaned subunits of multimeric protein complexes may be correctly folded so degradation must rely on a different mode of recognition, perhaps triggered by altered diffusion rates. Moving forward, studies of the SRP and KDEL receptor systems lay out an appealing roadmap to deeper understanding of contextual quality control systems. In both cases, many years of genetic and biochemical characterization were subsequently complemented with mechanistic detail from molecular structures and biophysical characterization using refined reconstitution assays. Such approaches will also be important in dissecting selection mechanisms during ER export. Looking forward, quantitative measurements of biophysical properties and kinetic parameters in the context of the crowded cellular environment will be the next step in dissecting how quality control pathways interpret and integrate contextual cues to protect the cell.

## Conflict of interest statement

Nothing declared.
